# Recent Perspectives on Cardiovascular Toxicity Associated with Colorectal Cancer Drug Therapy

**DOI:** 10.3390/ph16101441

**Published:** 2023-10-11

**Authors:** Monu Kumar Kashyap, Shubhada V. Mangrulkar, Sapana Kushwaha, Akash Ved, Mayur B. Kale, Nitu L. Wankhede, Brijesh G. Taksande, Aman B. Upaganlawar, Milind J. Umekar, Sushruta Koppula, Spandana Rajendra Kopalli

**Affiliations:** 1 Goel Institute of Pharmaceutical Sciences, Faizabad Road, Lucknow 226028, Uttar Pradesh, India; monukumarkashyap95@gmail.com; 2 Dr. A. P. J. Abdul Kalam Technical University, Lucknow 222001, Uttar Pradesh, India; akashved@gmail.com; 3 Smt. Kishoritai Bhoyar College of Pharmacy, New Kamptee, Nagpur 441002, Maharashtra, India; shubhadamangrulkar@yahoo.com (S.V.M.); mayur.kale28@gmail.com (M.B.K.); nitu.wankhede211994@gmail.com (N.L.W.);; 4 National Institute of Pharmaceutical Education and Research, Raebareli 229010, Uttar Pradesh, India; 5 SNJB’s Shriman Sureshdada Jain Collge of Pharmacy, Neminagar, Chandwad, Nadik 423101, Maharashtra, India; amanrxy@gmail.com; 6 College of Biomedical and Health Sciences, Konkuk University, Chungju-Si 27478, Chungcheongbuk Do, Republic of Korea; 7 Department of Bioscience and Biotechnology, Sejong University, Gwangjin-gu, Seoul 05006, Republic of Korea

**Keywords:** colorectal cancer, cardiotoxicity, chemotherapy, oxidative stress, apoptosis, 5-fluorouracil

## Abstract

Cardiotoxicity is a well-known adverse effect of cancer-related therapy that has a significant influence on patient outcomes and quality of life. The use of antineoplastic drugs to treat colorectal cancers (CRCs) is associated with a number of undesirable side effects including cardiac complications. For both sexes, CRC ranks second and accounts for four out of every ten cancer deaths. According to the reports, almost 39% of patients with colorectal cancer who underwent first-line chemotherapy suffered cardiovascular impairment. Although 5-fluorouracil is still the backbone of chemotherapy regimen for colorectal, gastric, and breast cancers, cardiotoxicity caused by 5-fluorouracil might affect anywhere from 1.5% to 18% of patients. The precise mechanisms underlying cardiotoxicity associated with CRC treatment are complex and may involve the modulation of various signaling pathways crucial for maintaining cardiac health including TKI ErbB2 or NRG-1, VEGF, PDGF, BRAF/Ras/Raf/MEK/ERK, and the PI3/ERK/AMPK/mTOR pathway, resulting in oxidative stress, mitochondrial dysfunction, inflammation, and apoptosis, ultimately damaging cardiac tissue. Thus, the identification and management of cardiotoxicity associated with CRC drug therapy while minimizing the negative impact have become increasingly important. The purpose of this review is to catalog the potential cardiotoxicities caused by anticancer drugs and targeted therapy used to treat colorectal cancer as well as strategies focused on early diagnosing, prevention, and treatment of cardiotoxicity associated with anticancer drugs used in CRC therapy.

## 1. Introduction

Colorectal cancer (CRC) is the leading cause of morbidity and mortality worldwide. CRC is the third most common cancer in women and the fourth most common cancer in men overall. In recent years, the prevalence of CRC has been rapidly rising worldwide. Globally, it was predicted that there would be 1.93 million newly diagnosed CRC cases and 0.94 million deaths from CRC in 2020, resulting in 10% of the total cancer incidence (more than 19.29 million new cases) and 9.4% of the total cancer-related deaths (9.96 million deaths). With an expected 419,536 fatalities for women and 515,637 deaths for men in 2020, CRC is the third highest cause of cancer-related death worldwide [1]. The only other cancer that causes more deaths from cancer is lung cancer. One in 17 males and one in 26 women will get CRC during their lives [2]. CRC is one of the most common malignancies, accounting for 10% of all cancer cases. It is the second most common cancer among women and the third most common cancer among males. It is the fourth leading cause of cancer death. It is more common in developed countries, accounting for over 65% of all cases [3]. The global burden of CRC is expected to rise by 60% by 2030, with more than 2.2 million new cases and 1.1 million cancer deaths [4]. Adenocarcinomas, together with adenosquamous and mucinous carcinomas, account for roughly 95% of CRC cases. Relative survival rates for CRC are 65% at 5 years and 58% at 10 years. The prognosis is not good regardless of the stage of the disease at diagnosis [5]. Moreover, 5.25 million people (5-year prevalence) are affected by CRC worldwide today, which is only slightly fewer than the 7.79 million instances of breast cancer. Increasing treatment options and great strides toward a better understanding of the pathophysiology of CRC have increased the overall survival of advanced CRC to three years. These treatments include endoscopic resection, surgical local excision, targeted therapy, radiation therapy, chemotherapy, and immunotherapy. 

Chemotherapeutic cardiotoxicity is any structural or function change in the cardiovascular system brought on by cytotoxic anticancer medication, as determined by a decrease in the left ventricular ejection fraction (LVEF). It may show symptoms that are acute, subacute, or oncology causes (recurrence or secondary neoplasm) are a more frequent cause of morbidity and mortality in patients who have received prior cancer treatment [6].

Concerns about an increased risk of cardiovascular disease (CVD) after a cancer diagnosis have recently been voiced [7,8]. This is because of shared risk factors, cardiotoxicity resulting from cancer treatment, and cancer biology-related mechanisms such as DNA damage, oxidative stress, free radical-induced mitochondrial dysfunction, etc. Chronic diseases connected to CVD and poor blood pressure control are known issues for those who have survived CRC. In relation to capecitabine, an oral prodrug of 5-fluorouracil (5fu), severe yet infrequently occurring adverse effects are considered cardiotoxic [7,8]. The most common symptom is chest pain similar to angina, and a variety of outcomes including cardiac arrhythmias, hypertension, hypotension, heart disease, coronary artery disease, cardiogenic shock, and sudden cardiac death can co-exist [9]. In most cases, patients stop receiving medication after the first cycle because of the aforementioned adverse events [10,11]. In this review, the common cardiotoxicity events that may occur during or after treatment for CRC will be discussed, along with their associated complications, biochemical pathogenesis, monitoring, and best management practices to prevent or lessen their effects.

## 2. Cardiovascular Complications Associated with CRC and mCRC Treatment

Cardiovascular (CV) complications during or following surgery for mCRC are uncommon, but do occur. A list of anticancer drugs causing cardiotoxicity is presented in Table 1. Perioperative CV difficulties such as acute myocardial infarction (MI), heart failure (HF), and CV death increase with patient age, underscoring the impact of frailty and the importance of optimizing CV condition in older patients with multiple comorbidities. Intravenous 5-fluorouracil (5-FU) and oral capecitabine are common first-line fluopyrimidines in chemotherapy for mCRC [12]. The pyrimidine analog 5-FU inhibits thymidylate synthase in an irreversible manner, making it an effective antimetabolite. When other cytotoxic medications like irinotecan and oxaliplatin are added to 5-FU, the survival rates for patients with metastatic disease improve. The reported incidence of cardiotoxicity due to 5-FU ranges from 1.2% to 18%. The most common clinical manifestation of 5-FU-induced cardiotoxicity is angina, followed by dyspnea, arrhythmias, and hypertension. ECG alterations such as ST segment changes, right bundle branch block (RBBB), arrhythmias such as atrial fibrillation (AF), pericarditis and pericardial effusion, acute myocardial infarction (AMI), apical ballooning syndrome, cardiac arrhythmias, heart failure (HF), and even mortality. Although the pathogenesis of 5-fluorouracil-induced cardiotoxicity is poorly understood, it has been suggested that direct myocardial toxicity, a procoagulant state, and endothelial injury leading to vasoconstriction are the key factors [13]. The most common method used is coronary vasospasm. Patients without occlusive macrovascular coronary disease (CAD) have been found to experience angina, ST changes, and a rise in troponin [14]. 

As an improved and safer alternative to 5-FU, capecitabine (a fluoropyrimidine derivative) was developed. It has the advantage of being orally administered and can be used in place of intravenous 5-FU or in combination with oxaliplatin in the first-line treatment of mCRC. Capecitabine is the oral prodrug form of 5-fluorouracil, and prolonged exposure of cancer tissue to capecitabine may result in less severe and frequent toxicity than 5-FU when administered alone or in combination with other chemotherapeutic drugs [15]. This is achieved by simulating the continuous administration of 5-FU while maintaining inadequate plasma levels. The bulk of these occurrences occur during the first cycle of treatment with capecitabine, oxaliplatin, and bevacizumab. Cardiotoxicity caused by bevacizumab can have effects that are both substance- and class-specific [16]. A hypersensitive reaction is the worst possible class effect, followed by life-threatening symptoms such as hypotension, asthma, fever, and hypoxia due to the massive release of cytokines. Bevacizumab’s two most common side effects, heart failure and hypertension, are both outcomes of this class [17]. Hypothesized molecular explanations for the development of hypertension or the worsening of pre-existing hypertension include variations in neovascularization, an imbalance in neurohormonal factors, endothelial dysfunction with lower vascular nitric oxide production, and renal failure. Hypertension is treatable, although its severity depends on the duration and intensity of exposure [18]. Hypertension typically manifests anywhere from one to six months after treatment has begun. Subarachnoid hemorrhage and encephalopathy are rare hypertensive emergencies [19], and only 5–36 percent of the population has severe hypertension (>200/110 mmHg). Active CV screening is thus highly recommended, particularly for people who have a high risk of developing CV illness. In the following sections, other major class of anticancer agents exhibiting cardiovascular toxicity used in CRC are discussed.

### 2.1. Monoclonal Antibodies

#### 2.1.1. Bevacizumab

Another class of medications that has shown promise as first- and second-line therapy for mCRC is monoclonal antibodies. Both bevacizumab and panitumumab have been approved by regulators for use against mCRC. Bevacizumab was the first angiogenesis inhibitor approved by the FDA for the treatment of mCRC [20]. This humanized IgG monoclonal antibody targets the circulating vascular endothelial growth factor A ligand (VEGF-A), which regulates angiogenesis in cancer cells. However, substance- and class-specific effects of bevacizumab-induced cardiotoxicity have been observed [21]. A hypersensitive reaction is the most lethal type of adverse event, characterized by life-threatening symptoms such as hypotension, asthma, fever, and hypoxia caused by the massive release of cytokines. Bevacizumab has several class effects, with heart failure and hypertension being the most common [22]. 

According to the manner in which the drug affects the cardiomyocytes, chemotherapeutic cardiotoxicity can be divided into type 1 or type 2 cardiotoxicity. Type 1 cardiotoxicity is irreversible because it results from the destruction of cardiomyocytes through necrosis or apoptosis. Type 2 cardiotoxicity may be reversible since it results from malfunction of the cardiomyocytes rather than cell death. The anthracyclines cause long-term cardiotoxicity, which includes cardiomyocyte loss and is classified as type 1 toxicity [23]. Understanding the cause of this cardiotoxicity has made it possible to create prophylactic measures that can prevent the onset of irreversible heart damage. While DNA damage is one of the main processes behind doxorubicin’s effectiveness in killing rapidly dividing cancer cells, free radical generation induced by the drug’s metabolism essentially causes it to be hazardous to cardiomyocytes. Particularly, NADH dehydrogenase in mitochondrial respiratory complex I reduces doxorubicin to produce a semiquinone radical that can combine with oxygen molecules to produce a superoxide radical. Redox cycling then causes the hydroxyl radical and hydrogen peroxide to be generated. By activating the apoptotic pathways, the reactive oxygen species generated by the metabolism of doxorubicin in cardiomyocytes ultimately result in cell death [24]. Doxorubicin induces apoptosis, which is partially mediated by p38 MAPK activation. Increasing the expression of Bcl-XL or Bcl-2 can protect against doxorubicin-induced cardiotoxicity even though there is evidence that early after doxorubicin exposure, there is initial upregulation of the antiapoptotic proteins Bcl-XL and Bcl-2 followed by decreases in their expression [23,25]. Understanding these cytoprotective mechanisms may offer insights into potential treatments that may lessen the toxicity of anthracyclines. Cardiomyocyte death is governed by the balance between those mentioned cytotoxic pathways.

Some potential molecular mechanisms for the onset or worsening of hypertension include endothelial dysfunction (with decreased vascular nitric oxide production), neovascularization rarefaction, neurohumoral variable discrepancy, and renal dysfunction [26]. Although hypertension is reversible, its effects depend on the duration and intensity of exposure. Furthermore, HF is classified as a third-class impact due to the direct detrimental effects on the myocardium, which may occur very infrequently in mCRC (1.3% of cases). Although the precise mechanisms at play are not known, bevacizumab-induced hypertension and the effect of VEGF suppression on myocardial repair and collateral vessel development may play a significant role [27]. 

#### 2.1.2. Cetuximab and Panitumumab 

Cetuximab and panitumumab are two examples of monoclonal antibodies that bind to the human epidermal growth factor receptor. Individuals with mCRC who have RAS and BRAF mutations can utilize them singly or in combination as first-, second-, or third-line therapies, respectively [28]. Third-line treatment with irinotecan alone, second-line treatment with 5-FU and irinotecan, and first-line treatment with 5-FU plus oxaliplatin or irinotecan (FOLFOX, FOLFIRI) are all possible applications. Most severe (2–5%) and deadly (0.1%) allergic reactions occur during the first hour after injection [29]. However, they can also occur hours later or after subsequent infusions. While studies on cetuximab are limited, it may increase the danger. On the other hand, panitumumab, a fully human monoclonal IgG2 antibody, was produced in a mammalian cell line. Panitumumab has been linked to venous thromboembolism, low magnesium levels, low potassium levels, dehydration, low blood pressure, and high blood pressure. Heart conditions requiring medical attention include palpitations/arrhythmias (25.8%), chest discomfort (8.1%), arrhythmias (4.8%), and dyspnea. Cetuximab and panitumumab have comparable CV toxicity profiles [30]. 

Tang et al. (2017) reviewed the assessment of the cardiac safety between cetuximab and panitumumab as single therapies in Chinese chemotherapy-refractory mCRC and concluded that cetuximab and panitumumab caused cardiotoxicity [31]. In the cetuximab group, there were still three patients with unspecified ST changes and three patients with QTc prolongation at 10 months after therapy, but in the panitumamab group, there were only two patients with nonspecific ST changes and four patients with QTc prolongation. The majority of the specific ST alteration and QTc prolongation brought on by cetuximab and panitumumab therapy may be reversible according to this result, however, the persistence of these abnormalities requires more investigation and care [31].

### 2.2. Tyrosine Kinase Inhibitors (TKIs)

#### 2.2.1. Regorafenib 

Regorafenib is a multi-kinase inhibitor that inhibits angiogenesis and tumorigenesis [32]. It inhibits the activities of VEGF receptors, RET, 1–3 PDGFR beta, TIE2, FGFR-1 and FGFR-2, DDR2, Eph2A, TrkA, BRAF, RAF-1, SAPK2, BRAFV600E, PTK5, and ABL [32]. A phase I trial with 53 advanced cancer patients examined the effects of repeated doses of regorafenib on cardiac function, specifically to formally assess the cardio safety of regorafenib. Regorafenib 160 mg/day administered to patients for 21 days, followed by a 7-day break showed no clinical significance except for four patients experiencing a 10 and 20% decrease in the left ventricular ejection fraction [33]. In another study, patients who had received fluoropyrimidines, irinotecan, oxaliplatin, bevacizumab, or are RAS wild-type and have EGFR antibodies were encouraged to take regorafenib, as recommended by the ESMO consensus guidelines for mCRC [34]. In patients with solid tumors, there is an increased risk of cardiovascular events caused by regorafenib [35]. Increased risk of hypertension and hemorrhage at all-grade was reported. 

#### 2.2.2. Novel Agents 

Trifluridine/tipiracil (TAS-102), a fluoropyrimidine, may be substituted for regorafenib in patients with mCRC who have previously been cured of cardiac disease with all active medicines including biologics. TAS-102 looks to be safe for the heart and has a good safety profile. Aflibercept, also known as Ziv-aflibercept or VEGF-Trap, is an intravenous injection of a human recombinant fusion protein that acts as a ligand trap to inhibit the activity of dissolved vascular endothelial growth factor A, vascular endothelial growth factor B, and placental growth factor [34]. Because of this, VEGF-dependent tumors are deprived of the oxygen and nutrients they need to grow. Aflibercept can be used as a second-line treatment for mCRC that has progressed or is resistant to an oxaliplatin-containing regimen in combination with FOLFIRI. It has been linked to manageability and a dramatically increased PFS [36]. Aflibercept, an inhibitor of vascular endothelial growth factor, most commonly results in hypertension; less frequently, HF, ATEs, and VTEs occur. Between 6.3% and 27.3% of the population have high-grade hypertension, whereas 16.7% to 51.4% have all-grade hypertension. Hypertension caused by aflibercept occurred in approximately 50% of patients during cycles 1–5 [37,38].

## 3. Molecular Signaling and Biomarkers Influencing Cardiovascular Toxicity in CRC

Mutation rates in protein kinases have been found to be quite high, according to data from tumor sequencing projects. According to one study, mutations in as many as 120 kinases (20% of the kinome) have been found in cancers [39]. While other protein classes (such as cell cycle regulators and pro/anti-apoptotic factors) can also be altered by oncogenic mutations, kinases have become the drug industry’s go-to target because of their importance in tumor progression and the ease with which inhibitors can be manufactured [40]. The first TKI to hit the market was imatinib (Gleevec, Novartis), which received FDA approval in 2001. In 2008, it raked in USD 3.67 billion in sales, making it by far the most successful TKI. Imatinib revolutionized the treatment of chronic myeloid leukemia (CML) [41]. All CML patients died within 5 years before the discovery of imatinib; now, 90% of patients are still alive 5 years after diagnosis. Thanks to these and other medications, cancers may now be viewed as a group of diseases that, while ultimately incurable, can be effectively managed over the course of many years, not unlike many other chronic illnesses [42]. Point mutations in the ATP-binding pocket of BCR-ABL are the most common cause of this. As a result of these mutations, imatinib’s affinity for binding to the ATP pocket is generally reduced in cancer cells [43]. Nilotinib and dasatinib, for example, are more effective drugs that block BCR-ABL and all drug-resistant variants of BCR-ABL, besides the missense mutation that produces BCR-ABL [44]. A list of anticancer drugs causing cardiotoxicity is shown in Table 1.

**Table 1:** List of anticancer drugs causing cardiotoxicity.

Sr. No.	Drug	Category	Mechanism of Anticancer Action	Type of Cardiotoxicity	Cancers Treated
1	Trastuzumab, Pertuzumab	Monoclonal Antibodies	HER2 inhibitors	LVSD, heart failure, orthostatic hypotension	Metastatic colorectal cancer, lung cancer, malignancies, myeloma [22]
2	Lapatinib, Sunitinib, Pazopanib, Sorafenib	Tyrosine kinase inhibitor	VEGF Inhibitors	Myocardial ischemia, LVSD, QT prolongation, arterial thromboembolic	Lymphoblastic leukemia, renal cell carcinoma, imatinib-resistant gastrointestinal stromal tumors, all types of chronic myeloid leukemia [32]
3	Imatinib, Dasatinib, Nilotinib, Bosutinib, Ponatinib	Tyrosine kinase inhibitor/ DNA damage	BCR-ABL Kinase Inhibitors	Accelerated atherosclerosis, peripheral artery disease, acute coronary syndrome, stroke, hypertension	Lymphoblastic leukemia, renal cell carcinoma, imatinib-resistant gastrointestinal stromal tumors, all types of chronic myeloid leukemia [32]
4	Carfilzomib, Bortezomib, Ixazomib, Doxorubicin, Epirubicin, Daunorubicin, Idarubicin, Mitoxantrone	Anthracyclines	Proteasome inhibitors, Oxidative stress, Topoisomerase II inhibitor	Myocardial ischemia, arterial hypertension, LVSD, heart failure, arrhythmias, QT changes, ventricular repolarization abnormalities	Gastrointestinal stromal tumors [28], acute leukemia’s, Hodgkin’s disease, non-Hodgkin’s lymphomas, breast and colorectal cancer [39]
5	Cyclophosphamide, Cisplatin, 5-Fluorouracil, Capecitabine	Alkylating agents, fluoropyrimines, fluoropyrimines	ROS production, DNA damage, Inhibit growth and metastasis	Acute heart failure (reversible), pericardial effusion, arrhythmias, myocardial ischemia, myocardial infraction	Blood, lung, breast, ovarian, endometrial, and bladder cancer [34], breast, colon, and other solid tumors [40]
6	Paclitaxel, Decetaxel	Taxane	Microtubule inhibition	Cardiomyocyte toxicity, bradycardia, LVSD, ventricular arrhythmias, myocardial ischemia	Cancers of the head and neck, prostatic, breast, bladder, and ovarian as well as Kaposi’s sarcoma, non-small-cell lung, and gastric adenocarcinoma [45]
7	Vincristine, Vinblastine, Vinorelbine	Vinca alkaloids	Microtubule inhibition	Myocardial ischemia	Lymphomas and leukemias [46]
8	Gemcitabine	Antimetabolite	Pyrimidine nucleoside antimetabolite	Pericardial effusion	Breast, bladder, pancreatic, and non-small-cell lung cancer [27]
9	Retinoic acid	Nutrient	Inhibiting the cell proliferation	Pericardial effusion, LVSD	Acute promyelocytic leukemia [40]
10	Arsenic Trioxide	Angiogenic	Interaction with ion channels	Prolonged QT Interval, Torsades De Pointes	Relapsing acute promyelocytic, leukemia [42]
11	Thalidomide, Lenalidomide	Angiogenic	Immunomodulator	Edema and sinus bradycardia, deep vein thrombosis	Multiple myeloma [45]
12	Interleukin 2	Cytokine	Block reproduction and spread of cancer cells	Hypotension, arrhythmias, myocardial ischemia, cardiomyopathy, myocarditis	Metastatic renal cell carcinoma and melanoma [47]

Abbreviations: LVSD, left ventricular systolic dysfunction; HER2, human epidermal growth factor receptor 2; VEGF, vascular endothelial growth factor Inhibitor; ROS, reactive oxygen species.

It is possible to create “intelligently” constructed inhibitors based on the knowledge of the structure of the pocket containing the mutation. In a case series involving 10 patients who received imatinib and went on to develop congestive heart failure, the very first report of hepatotoxicity with a small molecule TKI was made [45]. The initial study, which concentrated on the cardiotoxicity of a TKI, later revealed far more severe toxicity. In this investigation, patients with GIST taking sunitinib underwent serial assessments of the LV ejection fraction and biomarker findings (troponin I). In this study, 15% of patients experienced a reduction in LV ejection fraction or congestive heart failure, a total of 18% of patients. Cardiotoxicity has subsequently been linked to sorafenib, while the overall risk is unknown [47]. Only kinase inhibitors that specifically target critical kinases in the heart and vascular system are likely to be associated with cardiotoxicity, but it is important to emphasize that cardiotoxicity is not a class effect of kinase inhibitors [46].

### 3.1. Cardiotoxicity of the BCR-ABL1

**Figure 1:**
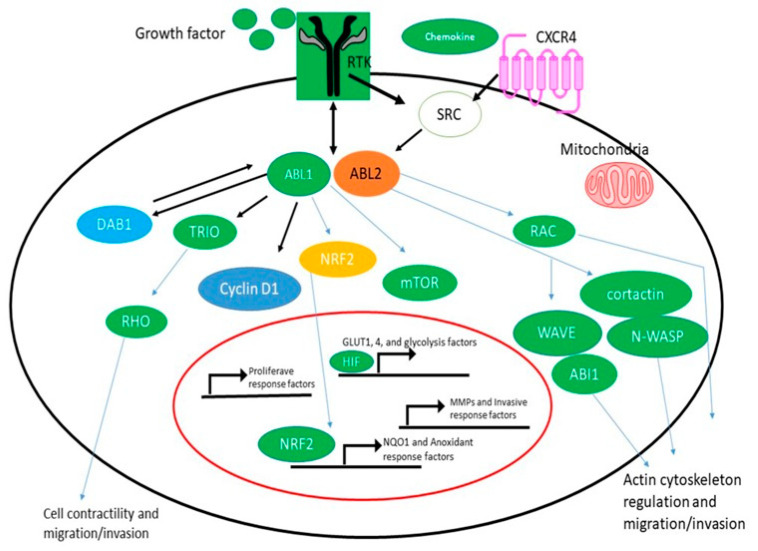
ABL Kinase Activation and Signaling in Solid Tumors. ABL kinases are activated by hyperactive receptor tyrosine kinases (RTKs), chemokine receptors, and SRC family kinases as well as by oxidative and metabolic stress. By activating multiple MMPs and actin-regulatory proteins such as RAC, cortactin, N-WASP, ABL interactor 1 (ABI1), and WAVE, activated ABL kinases promote cancer cell migration and invasion. ABL1 regulates cyclin D1 signaling downstream of the EPHB2 receptor to promote the activation of proliferative response factors in the intestinal epithelium and adenomas. In response to high fumarate levels, the ABL1 kinase becomes hyperactive in FH-deficient renal cancer cells (HLRCC); activated ABL1 promotes aerobic glycolysis via the mTOR-HIF1/pathway and also induces nuclear localization of the transcription factor NRF2 to induce the expression of NQO1 and other antioxidant response factors in HLRCC. NOTCH activation in the Apc^+^/D716 polyposis mouse intestinal epithelium promoted RBPJ-mediated transcription, leading to increased levels of DAB1, a substrate and activator of ABL kinases; activated ABL in colorectal cancer cells induced the tyrosine phosphorylation of TRIO on Y2681, leading to increased TRIO Rho-GEF activity.

The main causes of ABL kinase activation in solid tumors are the appearance and activation of either ABL1 or ABL2 as a result of amplification, enhanced expression of gene, increased protein levels, and enzyme activity in response to being induced by oncogenesis, chemokines, oxidative disturbances, and the inhibition of harmful transcription factors [44]. According to the Cancer Genome Atlas and other large-scale sequence endeavors, amplification of ABL, alterations in somatic cell DNA, and increased expression of mRNA have all been discovered in a variety of solid tumors. Twenty-four percent of hepatocellular carcinomas and, to a lesser extent, cancer of the uterine endometrium (twenty percent), invasive breast cancer (nineteen percent), and adenocarcinoma of the lung cell (15 percent), have been reported to have changes in ABL2 [48]. ABL2 mutations, which are more frequent in ABL2 than ABL1, have been found in more than 80% of patients. These results agree with previous data showing that ABL2 expression is increased in aggressive forms of breast, colorectal, hepatic, renal, and gastric tumors [49]. By connecting NOTCH activation to the phosphorylation of TRIO (pY2681), which results in higher TRIO Rho-GEF function and a commensurate rise in the Rho GTP level, recent research has shown a specialized role for ABL kinases in promoting colorectal cancer cell metastasis and invasion. NOTCH activation in the intestinal mucosa of pc+/D716 polyposis mice was produced by the homozygous deletion of AES, which accelerated RBPJ-mediated transcription and increased the amounts of DAB1, a substrate and ABL kinase activator. TRIO Rho-GEF activity was upregulated by tyrosine phosphorylation at Y2681 in colorectal cancer cells expressing active ABL (Figure 1) [50].

Invasion, extravasation, and metastasis were all aided by Rho activation in colorectal cancer cells. Notably, tumor size was unaffected by ABL kinase inhibition in Apc/Aes combination knockout mice, but the incidence of invasion and intravasation was drastically reduced. Based on these results, ABL kinases may function as a bridge between the activation of extracellular receptors and Rho signaling in certain malignancies. Rho activation is needed for cell dispersal, tubulogenesis, migration, and invasion, and recent investigations have shown that ABL kinases link Rho activation to the ligand-activated MET tyrosine kinase [50]. 

Here, we show how ABL regulates these processes in solid tumors, with a focus on research that has used knockout/knockdown strategies or specific allosteric inhibition to target the ABL kinases, as opposed to the use of ATP-competitive TKIs that inhibit multiple tyrosine kinases [44]. It has been demonstrated that TKIs like imatinib, dasatinib, and dasatinib have inhibitory and, in certain circumstances, stimulatory effects on cancer cell motility, survival, and proliferation. The cellular reactions to these medications, however, cannot be solely attributed to the suppression of ABL kinases because these chemicals also target a range of other kinases and certain non-kinase enzymes.

Yllka Latifi et al. [51] found that the BCR-ABL1 tyrosine kinase inhibitor ponatinib was associated with adverse cardiovascular events. A total of 33% of wild-type and 45% of ApoE−/− rats had segmental left ventricular wall motion abnormalities and patchy perfusion deficits after receiving ponatinib, but the angiograms of both groups showed a normal coronary artery morphology. Instead, intravital microscopy was used to see platelet aggregation and nets connected to leukocytes and endothelial cells, and immunohistochemistry was used to spot global microvascular angiopathy to come to this conclusion. These results suggest that the TKI ponatinib causes a novel form of vascular toxicity characterized by VWF-mediated platelet adhesion and subsequent microvascular angiopathy that modifies ischemic wall motion. Interventions known to diminish the size of the VWF multimer [52] can help lessen the effects of these processes. These results suggest that the TKI ponatinib causes a novel form of vascular toxicity characterized by VWF-mediated platelet adhesion and subsequent microvascular angiopathy that modifies ischemic wall motion. Interventions known to decrease VWF multimer size can lessen the effects of these processes [53].

### 3.2. Colorectal Tumors and Epidermal Growth Factor Receptor Signaling

The ErbB family of receptor tyrosine kinases comprise ErbB1, 2, 3, and 4. ErbB1, also referred to as EGFR, is a standard constituent of the ErbB clan, featuring a tyrosine kinase that becomes active upon ligand binding. The EGFR, a transmembrane glycoprotein with a molecular weight of 170 kDa, is comprised of three distinct regions: an intracellular tyrosine kinase (TK) domain, a transmembrane lipophilic region, and an extracellular ligand-binding domain [54]. Under typical physiological conditions, the ErbB receptors are indispensable for transmitting signals that govern cell division, motility, proliferation, and apoptosis. A multistep kinase cascade, which triggers the activation of MAPKs, is initiated by the activation of Ras. Due to the significance of the EGFR axis in the development of cancer and advancement of tumors, the potential of EGFR expression as a prognostic biomarker in patients with colorectal cancer (CRC) has been examined [54].

Although to different degrees, EGFR expression was observed in up to 82% of colorectal carcinomas. The assessment of EGFR expression in tumor tissues has been conducted by examining gene amplification, mutation, increased mRNA transcripts, or raised protein levels. In colorectal cancer (CRC), it has been demonstrated that there exists a connection between elevated levels of EGFR expression and the invasion of tumors. Furthermore, a significant correlation has been observed between high EGFR expression and the TNM cancer stage at the time of diagnosis [55]. The most advanced stages of clinical development for CRC comprise monoclonal antibodies such as cetuximab and panitumumab (phase III trials), along with low molecular weight TKIs like gefitinib and erlotinib (phase II studies). The active ingredients comprise panitumumab (ABX-EGF or Abgenix^®^ (Fremont, CA, USA), total human IgG2) and cetuximab (Erbitux^®^, Oxford, UK, chimeric IgG1). Single medicines or in combination with chemotherapy are currently being investigated in phase III clinical trials for the treatment of recurrent and first-line metastatic colorectal cancer (mCRC). The treatment of mCRC that was resistant to irinotecan was authorized by the U.S. Food and Drug Administration in 2004 through the use of cetuximab plus irinotecan [56]. Data from various clinical studies suggest that there is no definitive correlation between the level of EGFR expression and the efficacy of second-line cetuximab therapy in treating tumors.

According to two recent phase II trials involving panitumumab in refractory metastatic colorectal cancer (mCRC), patients exhibiting robust EGFR expression (as confirmed by IHC, with 10% of tumor cells) achieved response rates (RR) that were comparable to those with low or negative EGFR expression (with 9% of tumor cells). Furthermore, the responsiveness of tumors to gefitinib plus chemotherapy was not associated with the levels of EGFR expression in 31 individuals with mCRC. While extending cancer patient survival, erbB2 antagonists also disrupt the heart’s homeostatic mechanisms [57]. ErbB4’s preferred coreceptor, ErbB2, is activated by NRG-1. This growth factor, bearing a resemblance to epidermal growth factor (EGF), is secreted by endothelial cells present in the endocardium and myocardial microcirculation. It facilitates intercellular communication in the ventricle. Following the identification of the gene deletion of the NRG-1 or ErbB receptors as the cause of dilated cardiomyopathy or cardiac malformation, research was conducted to explore the impact of NRG-1 on cardiac cell and tissue responses. NRG-1 was discovered to enhance cell–cell adhesion and induce cardiomyocyte hypertrophy by augmenting the cardiomyocytes’ resistance to apoptotic cell death [58]. The involvement of NRG-1 in the nitric oxide synthase-dependent desensitization of adrenergic activation and angiogenesis is believed to be significant. 

**Figure 2:**
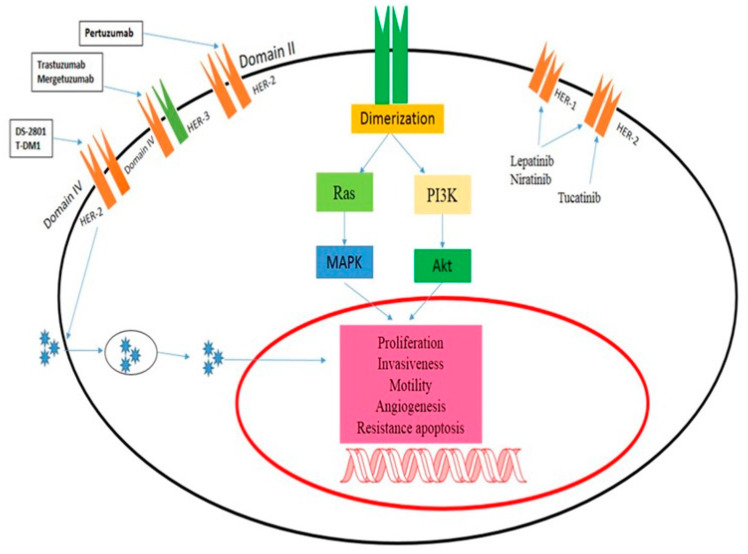
Mechanism of action of ErbB2-targeted drugs. Panitumumab inhibits Domain II in HER-2, trastuzumab-induced heart failure, and the cardiomyopathy brought on by ErbB2 deletion have led many to the conclusion that trastuzumab induces cardiotoxicity by impeding the physiological functions of ErbB2 in the heart.

New research by Bersell et al. suggests that NRG-1 injections in adult mice can stimulate myocardial regeneration and increase cardiomyocyte cell cycle activity [59]. This leads to improved functionality after myocardial infarction. Additionally, an in vitro “cardio protective program” was launched [60]. The NRG-1/ErbB system plays a vital role in the heart, and there is a striking similarity between the cardiomyopathy caused by ErbB2 deletion and trastuzumab-induced heart failure. This has led to the belief that trastuzumab causes cardiotoxicity by obstructing the natural functions of ErbB2 in the heart. Although fair and compelling, this theory still requires more research. The negative side effects of ErbB1 inhibitors are minimal. The most common side effect of EGR inhibition is skin rash, possibly caused by the presence of EGFRs in the epidermis. Diarrhea is another frequent side effect in individuals treated with TKIs, but not in those treated with mAbs. Gefitinib-related interstitial pneumonitis stands as the sole recorded serious hazard linked to these drugs [61]. Erlotinib and gefitinib appear to exhibit a low occurrence of cardiotoxicity when employed solely as inhibitors of ErbB1. The cardiotoxic effects observed in ErbB1-targeted therapies may indirectly indicate reduced ErbB2 signaling, as ErbB2 often forms dimers with ErbB1 upon interaction with ligand-bound ErbB1 (Figure 2) [62].

### 3.3. VEGFRs

Recent interest in the vascular endothelial growth factor (VEGF) pathway can be attributed to the discovery that inflammation plays a role in the development of colorectal cancer. Vascular endothelial growth factor (VEGF) is often considered the main mediator of tumor angiogenesis [63]. Endothelial cells (ECs) generate vascular endothelial growth factor receptor 2 (VEGFR2). When VEGFR2 is activated, ECs migrate, multiply, and have a better chance of survival. Therefore, bevacizumab (an anti-VEGF antibody) was approved for the treatment of human cancer as a result of studies showing that such approaches could inhibit tumor growth [64]. Numerous animal models have established the significance of VEGF in post-natal cardiac homoeostasis and proper heart development. VEGF promotes the development of rat neonatal cardiomyocytes from mouse embryonic stem cells through the expression of VEGF receptors. In addition, VEGF promotes cell proliferation and turnover in rat cardiomyocytes through activating mitogen-activated protein kinase (MAPK) pathways. As a protective mechanism against cardiac stressors, cultured human cardiomyocytes secrete VEGF in response to inflammation, ischemia, and hypertension [65]. VEGFI-induced cardiotoxicity can be reversed, at least in part. 

One study analyzed prospective echocardiographic and biomarker data from 90 individuals using sunitinib for RCC. A total of 9.7% of patients had a decline in LVEF of 10% to 50% or more from the baseline. The majority of patients who eventually acquired LVSD did so early in treatment. Up to 33 weeks of follow-up, treatment with sunitinib dose decrease and cardiac medications (variously including ACE inhibitor, blocker, and dihydropyridine calcium channel blocker) improved LVSD function. A randomized controlled trial involving patients with imatinib-resistant gastrointestinal stromal cancer showed that sunitinib reversed VEGFI-associated LVSD [66]. Slowly worsening congestive heart failure was seen in 8% of patients over a 24-week follow-up period, while a decline in LVEF of less than 10% was seen in 28% of patients. However, after adjusting the dosage and receiving heart failure treatment, all patients who developed congestive heart failure saw an improvement in LVEF and symptom alleviation. 

**Figure 3:**
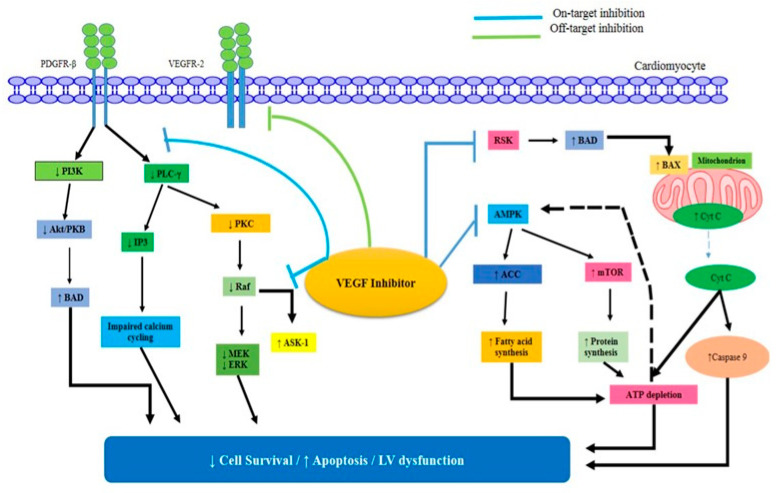
The VEGF inhibitors’ mechanisms of direct myocardial damage. The pro-apoptotic factor BAD may become active in response to the inhibition of ribosomal S6 kinase (RSK). As a result, BAX is activated, and the mitochondria release Cyt C.

Cardiomyocyte hypertrophy and aberrant mitochondrial topologies were seen in the endomyocardial biopsies of two heart failure patients, although there were no signs of apoptosis or fibrosis. Whorls, other degenerative alterations, and larger mitochondria were seen in the cardiomyocytes of sunitinib-treated animals. Endomyocardial biopsies from three patients with VEGFI-associated cardiotoxicity showed similar mitochondrial abnormalities but no apoptosis or fibrosis. Repeat biopsies performed after drug withdrawal and the beginning of heart failure medicines showed a marked improvement in mitochondrial and overall cardiac performance [67]. Activation of the AMPK/mTOR/ribosomal S6 kinase/autophagy pathway by trimetazidine improves cardiomyocyte viability and rescues sunitinib-treated animals from cardiotoxicity. Improved cardiac energy metabolism is achieved by the selective inhibition of mitochondrial long-chain 3-ketoacyl-CoA thiolase by trimetazidine. Because of this enhanced energy turnover, the myocardium is more resistant to hypoxia, as shown by the effects of VEGF on AMPK activation (Figure 3) [68].

### 3.4. PDGF

Angiogenesis is characterized by several signaling pathways and a balanced ratio of pro- and antiangiogenic substances [69,70]. The “angiogenic switch”, an imbalance between pro- and antiangiogenic factors that results in an increase in the nutrition supply essential for tumor growth, is present at the earliest stages of malignancy. Tumor angiogenesis is a mechanism necessary for the development and progression of CRC. PDGF signaling promotes lymphatic angiogenesis and subsequent lymphatic metastasis by recruiting pericytes to the vasculature, activating proangiogenic factors, increasing endothelial cell proliferation and migration, and stimulating lymphatic angiogenesis. Modification of signaling by the PDGFR family is critical to the progression of colorectal cancer. In most cases, CRC is associated with the overexpression of PDGFR in tumors and stromal cells around tumors. PDGFR overexpression in CRC has been linked to poor survival, angiogenesis, invasion, metastasis, and target-associated treatment [69,70]. 

More recently, it was shown that PDGF-BB’s role in CRC is connected to the proliferation of pericytes within tumors. Endothelial cell survival is promoted, vascular function is regulated (for example, by modulating vessel width and permeability), and mechanical support and stability are maintained thanks to pericytes. PDGFR-, which is widely expressed on VSMCs and pericytes, is the primary receptor for the PDGF-BB homodimer. The use of angiogenesis inhibitors has revolutionized the treatment of several forms of cancer. As tumors expand beyond the capacity of the existing vasculature to supply oxygen, the cancer cells generate proangiogenic cytokines such as vascular endothelial growth factor (VEGF). Angiogenesis, the formation of new blood vessels, is essential for the development and metastasis of solid tumors, and is regulated by cytokines that either promote or inhibit angiogenesis [71].

### 3.5. BRAF/Ras/Raf/MEK/ERK Pathway

**Figure 4:**
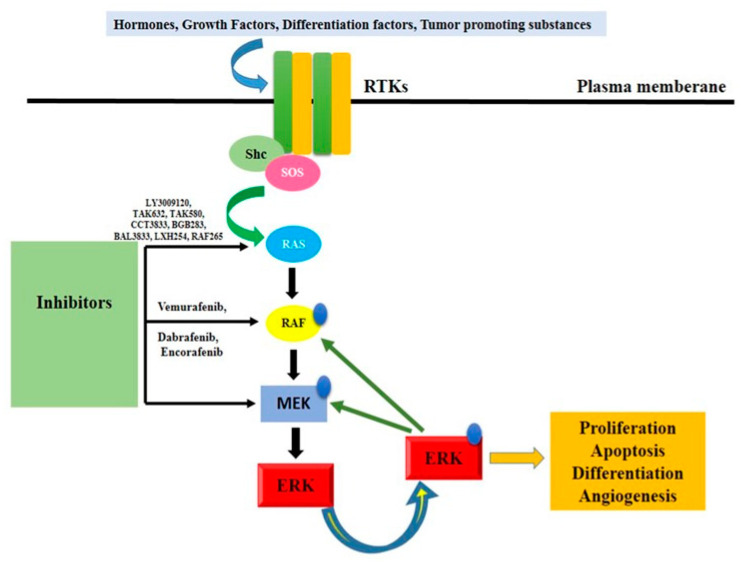
Ras/Raf/MEK/ERK signaling pathway activation. It denotes a critical protein that is phosphorylation-controlled. Rtk, receptor tyrosine kinase, or RTK mammal son-of-sevenless; MAPK, mitogen-activated protein kinase kinase; ERK, extracellular signal-regulated kinase; Shc, Src homology 2 domain-containing protein.

There is conclusive evidence that the RAS signaling cascade contributes to tumor growth. Point mutations in any of the cascade proteins are associated with either tumorigenesis (in the case of RAS and RAF mutations) or a poor prognosis (in the case of MEK and ERK mutations). It is worth noting that antagonistic driver mutations exist at every stage of the process [72]. This section discusses the mutation hotspots for each part of the signaling cascade. Ras/RAF/MEK/ERK signaling cascades (Figure 4) involve RAF as a critical direct effector of oncogenic Ras mutants and as a prominent target of oncogenic alterations. RAF, the first kinase in this pathway, has been studied extensively because of its potential as a drug target for cancer treatment. Vemurafenib, dabrafenib, and encorafenib, first-generation RAF inhibitors, were developed and utilized as monotherapies or in conjunction with MEK inhibitors to treat cancers harboring the BRAF (V600E) mutation [73]. These drugs had promising early results in treatment, but their effectiveness quickly waned as drug resistance spread. Cancer cells reactivate this pathway in response to drug treatment in two distinct ways, as revealed by mechanistic studies: (1) increasing the amount of active Ras in the cell, which results in paradoxical activation of ERK signaling, and (2) alternatively splicing BRAF (V600E) to produce variants with a truncated N-terminus, which enhances BRAF (V600E) homodimerization and reduces drug affinity. It is interesting to note that RAF inhibitors become addictive to drug-resistant cancer cells, and that discontinuing treatment with these drugs delays the evolution of resistant malignancies [72]. This can be explained by the concept of a “sweet spot” for hyperactive Ras/RAF/MEK/ERK signaling-driven cancer growth.

Particularly, adequate ERK signaling is necessary for cancer cell growth, but excessive ERK signaling will cause cell death or senescence, making it harmful to cancer cells. More ERK signaling can be found in drug-resistant cancer cells than in drug-sensitive ones. The ERK signaling of drug-resistant cancer cells is reduced during drug treatment, allowing them to thrive, but is halted after medication withdrawal or a “drug holiday” [73]. The paradoxical effect was a serious drawback of first-generation RAF inhibitors that not only diminished their effectiveness but also led to the development of secondary malignancies. Numerous investigations utilizing cultured cardiomyocytes have demonstrated that the Raf/MEK/ERK pathway is either essential for hypertrophy, has an antihypertrophic effect, or regulates the induction of hypertrophic genes but not actual cell growth. Using transgenic mice, Bueno and coworkers demonstrated that localized generation of activated MEK in the heart caused considerable concentric hypertrophy [69]. Heart hypertrophy and ventricular contractility were both preserved in mice with activated MEK transgenics. Furthermore, activated MEK transgenic mouse heart tissue was resistant to apoptotic stimuli in some areas. This anti-apoptotic effect may result from ERK’s ability to inhibit caspase-9. Understanding the pathogenic control of the Ras/Raf/MEK/ERK cascade in muscle cells is crucial due to the established involvement of this system in the development of forelimb muscle, arteriogenesis, heart failure, and myocardial infarction. 

The Ras/Raf/MEK/ERK cascade is not necessarily linear, and this is an important point to keep in mind. Muscle progenitor cell migration, for instance, is controlled by BRAF and is independent of ERK activity. Due to reprogramming of this signaling cascade, deregulated cells may become resistant to specific Raf/MEK inhibitors, as is known from cancer therapy. The fact that variations in the activation or inhibition of the master gatekeepers MEK1/2 did not always result in the predicted patterns of phosphorylation suggests that the regulation of this well-studied pathway is far more nuanced than previously thought. It is possible to expect unfavorable cardiovascular effects from pharmacological manipulation of the Ras–RAF–MEK–ERK pathway because of its pivotal role in cardiac and vascular physiology. In fact, BRAF inhibitor/MEK inhibitor medication is associated with LVSD, systemic hypertension, atrial arrhythmia, QT interval prolongation, and venous thromboembolism [72,74].

### 3.6. mTOR

Mutations in the APC gene (adenomatous polyposis coli) are common causes of colorectal cancer. mTOR is essential for the regulatory network to detect growth signals from nutrients and regulate cell proliferation. The use of mTOR inhibitors in the treatment and prevention of tumor growth, particularly in CRCs, has been the subject of much speculation. Intense research has been conducted to develop potent and efficient compounds along the mTOR pathway [75].

Breast cancer, like many others, has an 80% survival rate. These developments could be attributed, in part, to chemotherapeutics such as doxorubicin (DOX), an anthracycline antibiotic initially discovered from *Streptomyces peucetius*. Childhood cancer survivors are especially affected by the growing awareness of the long-term side effects of chemotherapeutics. Cardiotoxicity is one of the most dangerous effects of chemotherapy and is defined as a drop in the left ventricular ejection fraction (LVEF) of more than 10% to a value lower than 50%. DOX cardiotoxicity is particularly cardiotoxic, causing congestive heart failure in 5% of patients, while its incidence varies widely depending on the dose (from 3% to 18%). To lessen the risk of cardiotoxic consequences, the maximum DOX dose for a lifetime has been lowered to 450 mg/m^2^ [76]. Cardiotoxicity caused by DOX varies by sex in both humans and animal models, with prepubescent and postmenopausal women more likely to have adverse effects.

**Figure 5:**
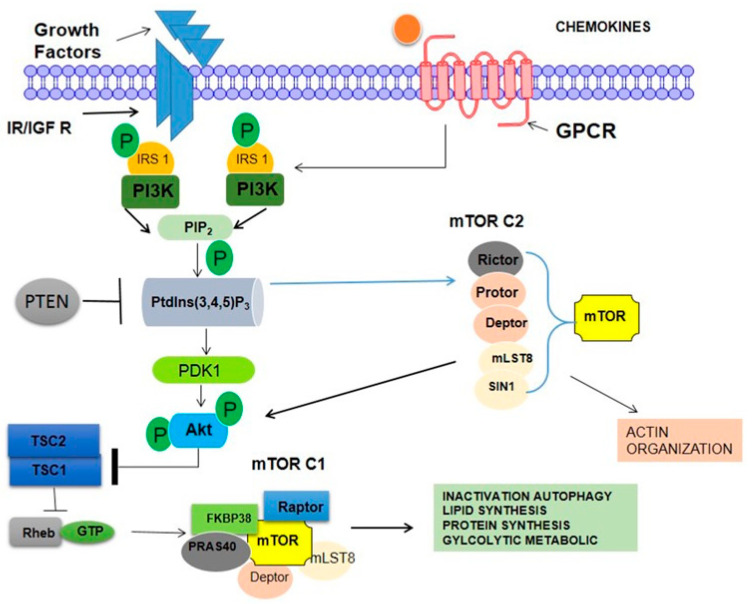
Mammalian target of rapamycin (mTOR) complexes (mTORCs) and the phosphoinositide 3-kinase (PI3K)/Akt signaling network. mTORC1 comprises regulatory-associated protein of mTOR (RAPTOR), proline-rich Akt substrate 40 kDa (PRAS40), mammalian lethal with Sec13 protein 8 (mLST8), and DEP domain-containing mTOR-interacting protein (DEPTOR), once TSC2 is phosphorylated by Akt, the GAP activity of the TSC1/TSC2 complex is repressed, allowing Rheb to accumulate in a GTP-bound state. As a consequence, Rheb–GTP upregulates the protein kinase activity of mTORC1. mTORC2 is involved in the spatial control of cell growth via cytoskeletal regulation.

There is more detailed discussion elsewhere on the incidence, risk factors, timing, and outcomes of DOX treatment for cancer patients. DOX accumulates in the heart through binding to cardiolipin in the inner mitochondrial membrane. DOX clearance from the myocardium is substantially slower than clearance from plasma, which may explain why the heart is more vulnerable to its effects. There are a number of hypothesized ways by which DOX exerts its cardiotoxic effects. Topoisomerase II inhibition is thought to be the major mechanism by which DOX exerts its anticancer effects by inducing apoptosis and DNA double-strand breaks. Recent evidence suggests that topoisomerase II contributes to the cardiotoxicity of DOX, which may lead to DNA damage and mitochondrial dysfunction [53], despite the fact that topoisomerase II is absent in cardiomyocytes. Mitochondria take up almost half of the cardiomyocytes’ total volume. They play a vital role in generating energy via the sequential pathways of the tricarboxylic acid (TCA) cycle, electron transport chain, and oxidative phosphorylation (Figure 5) [43].

### 3.7. PI3K Pathway

The vast majority of oncology medication research and development efforts are currently focused on inhibiting various steps along the PI3K pathway. We therefore go over this pathway, any possible targets for heart cancer, and the present state of therapeutic research in this field. Numerous malignancies have alterations or amplifications in every component of the PI3K pathway, making it a promising target for cancer treatment. Cellular responses in cancer are triggered by mutated or amplified RTKs (with the PI3K pathway playing a pivotal role in facilitating these reactions). Some examples are Kit, PDGFRs, and Met, in addition to epidermal growth factor receptor and human epidermal growth receptor 2. The PI3K p110 isoform is also highly mutated in many cancers including 27% of breast and 23% of endometrial cancers [53,77]. Three missense mutations within the kinase and helical domains of p110 cause the kinase to be permanently active. p110 is also upregulated in several forms of solid tumors. Mutations in the PIK3CA gene, which codes for the p110 subunit, were found in 6% of nonmalignant lesions in a recent analysis of colorectal polyps and CRC, suggesting that these genetic changes may be initial events in the development of CRC. Similarities between cancer signaling and cardiomyocyte hypertrophy/survival signaling are best demonstrated by the PI3K/PDK1/Akt/mTOR/S6K pathway [73,77].

Although mTOR inhibitors have been shown to be effective in hypertrophic experimental models and to be generally well-tolerated in transplant patients over the long-term, there are concerns that the inability of mTOR to react adequately to the energy prestige of the cardiomyocyte when used in combination with therapies that target other components of the passageway will increase the toxicity [69].

### 3.8. p38 Mitogen-Activated Protein Kinase

Since p38 mitogen-activated protein kinase (MAPK) was discovered to play a critical role in the production of the pro-inflammatory cytokine’s tumor necrosis factor and interleukin-1, it has been the target of kinase inhibitor development for the treatment of inflammatory illnesses. The p38 MAPK pathway, in concert with other signaling cascades such as JNK, ERK, AMPK, and PI3K, controls the equilibrium between cell viability and cell death, therefore influencing the development of different malignancies. Tight control of survival/death signals by p38 MAPKs during tumor growth can result in competing molecular functions (Figure 3) [72]. It is true that p38 MAPKs serve a dual role; depending on the pathway they are activated through, they can either promote cell survival or trigger cell death. SB-239063, a specific p38 MAPK inhibitor, has been proven in recent preclinical trials to reduce endothelial dysfunction, angiotensin II-induced hypertension, cardiac hypertrophy, and atherosclerotic plaque inflammation. Forty-nine recent investigations showed that low-dose p38 MAPK inhibitor treatment for three months, in patients with cardiovascular disease who are already receiving statin therapy, reduced C-reactive protein and improved vascular responsiveness [78]. 

The confluence of clinical disciplines is a guiding principle in cardio-oncology. Providers of cardio-oncology care must be familiar with the whole range of cardiology, oncology, and hematology management. Recommendations are made for the cancer treatment that is both the most ethical (from a CVD standpoint) and the most efficient (from an oncological one). Another crucial component of cardio-oncology management is the adjudication of CV events that occur in patients receiving active therapy. These developments may occur at somewhat different times. A new risk assessment is advised to determine whether various long-term outcomes are affected by environment factors, stresses (such as acute viral infections), patient-related CVRF, some cancer medications’ irreversible harmful effects on the cardiovascular system, cardiac or vascular injuries, and other environmental factors [79,80]. 

## 4. Principle Techniques Used for Monitoring Cardiotoxicity in Clinical Practice

To reduce the risk of cardiotoxicity in cancer patients, it is important to promote a healthy lifestyle (regular exercise, a nutritious diet, and giving up smoking) as well as to identify and treat cardiovascular risk factors like dyslipidemia, elevated glycated hemoglobin, and hypertension [80]. Using less cardiotoxic regimens and lowering the cumulative dose of cardiotoxic drugs are two other ways to lessen cardiotoxicity [81]. It is possible to prevent or minimize cardiotoxicity by using drugs with a cardioprotective effect. 

The recent international definition of CTR-CVT is the outcome of the necessity to standardize these concepts, which has been repeatedly expressed and acknowledged. The accepted definitions for cardiomyopathy, heart failure (HF), myocarditis, vascular toxins, hypertension, cardiac arrhythmias, and prolonged corrected QT interval (QTc) will be the main focus of this work. Other CTR-CVT definitions such as those for pericardial and valvular heart disorders (VHDs) follow those that are used for the general cardiology population. The descriptive term cancer therapy-related cardiac dysfunction (CTRCD), which encompasses the wide range of potential presentations and the etiological connection with a wide range of different cancer therapies such as chemotherapy, targeted agents, immune therapies, and radiation therapy. A list of cancer therapy-related cardiovascular toxicity definitions is provided in Table 2. 

Pre-treatment of the optimum way to perform a CTR-CVT risk assessment is the incorporation of numerous risk indicators to calculate patient-specific risk. These developments may occur at somewhat different times. A new risk assessment is advised to determine various long-term dynamics of CV health after the cardiotoxic cancer treatment is concluded. Clinical history, physical examination, ECG, cardiac biomarker analysis and transesophageal echocardiography (TTE) should all be included in the cardiac assessment performed on pregnant cancer patients before they begin chemotherapy. TTE at baseline and follow-up should be interpreted in light of physiological changes to pregnancy-related hemodynamics. In a healthy pregnancy, an increase in cardiac output from the first trimester to 80–85% over baseline by the third trimester is caused by an increase in stroke volume, heart rate, and pre-load blood volume as well as a decrease in systemic vascular resistance. The following agents are used as preventative measures because of their cardioprotective properties.

### Statins Reduce Anthracycline-Induced Heart Failure and Cell Damage

Statins, or 3-hydroxy-3-methylglutaryl-coenzyme A(HMG-CoA) reductase inhibitors, are widely regarded as the first-line treatment for lowering the risk of adverse cardiovascular events. Statins lower serum cholesterol by blocking HMG-CoA reductase, which reduces the amount of new cholesterol that is produced and increases the removal of low-density lipoprotein from the bloodstream. ECG, structural and functional evaluation (echocardiography, biomarker testing), risk classification, and medication of cardiovascular risk factors are all part of the first patient evaluation. Biomarkers (troponin +/− pro-BNP) should be measured at the beginning and end of treatment (in the case of TKIs, every three months), and transthoracic echocardiography should be performed periodically throughout the course of treatment [82,83]. A number of anterogenic process components are inhibited by endothelial nitrous oxide (NO), for instance, regulating endothelial–leukocyte interaction, preventing platelet aggregation and vascular smooth muscle proliferation and controlling vascular relaxation. Statins prevent the synthesis of the isoprenoid intermediates farnesyl pyrophosphate (FPP) and geranyl pyrophosphate (GGPP) in order to reduce the production of cholesterol. Important Rho protein components are FPP and GGPP. The stability of the eNOS messenger ribonucleic acid (mRNA) and eNOS phosphorylation can both be decreased by the active form of Rho, which in turn lowers eNOS production and activity. By preventing Rho from being prenylated, statins increase the stability of eNOS mRNA [84]. The major way that statins exert their pleiotropic effect is by inhibiting isoprenoids, which act as lipid attachments for intracellular signaling molecules. The mevalonate pathways’ rate limiting enzyme is HMG-CoA. Mevalonate is required for the production of isoprenoid intermediates, which are crucial for the activation of small GTP-binding proteins like Rac, Ras, and Rho for membrane translocation. It is also possible that mevalonate plays a significant role in mediating the pleiotropic effects of statins [85].

Cardiology consultation, increased monitoring frequency, and medication changes are all necessary for patients with decreased LVEF or elevated biomarkers at the baseline or throughout therapy. Testing of LV function following therapy is recommended according to the 2020 guidelines from the European Society of Cardiology and the European Society of Medical Oncology, even if the total dose is 300 mg/m^2^. Following therapy and at six months post-completion, an echocardiogram with GLS should be performed, as recommended by the 2014 consensus recommendations of the American Society of Echocardiogram and the European Association of Cardiovascular Imaging. 

**Figure 6:**
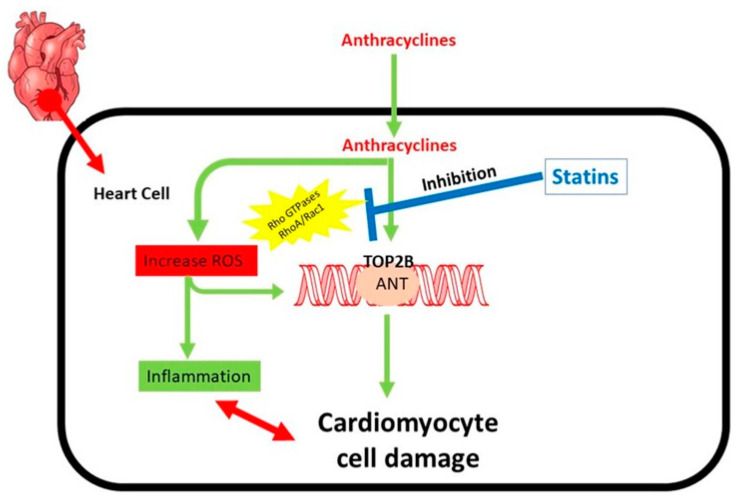
Mechanism involved in relation to the cardiotoxicity caused by anthracyclines. The main contributors to the cardiotoxicity caused by anthracyclines are regulated by rho GTPases. Redox cycling and Fenton’s reaction are two ways that anthracyclines cause reactive oxygen species (ROS). ROS are the cause of inflammation and generate oxidative DNA damage. Anthracyclines also inhibit type II topoisomerases (TOP2), leading to extremely cytotoxic DNA double strand breaks (DSBs), which compel a pro-apoptotic DNA damage response and may ultimately cause cardiomyocyte cell death. RhoA and Rac1 engage in the regulation of the inflammatory process.

Doxorubicin and daunorubicin are cytostatic anthracyclines that were discovered in fungi of the genus streptomyces. Doxorubicin exhibits a broad range of activity against both solid tumors and hematological malignancies. It is used to treat non-Hodgkin’s lymphoma, lymphocytic, and myelogenous acute leukemias. Cardiotoxicity brought on by anthracyclines likely has several contributing factors. One of the most widely researched methods by which anthracyclines have been postulated to produce cardiotoxicity is free radical-mediated myocyte destruction. About 20% to 25% of patients with colorectal cancer have elevated levels of the human epidermal growth factor (HER2), which is linked to a poor prognosis. Trastuzumab, according to the study, increases the clinical benefit of chemotherapy for metastatic colorectal cancer with overexpressed HER2. However, there was a 27% incidence of cardiac dysfunction in the group receiving anthracycline, cyclophosphamide, and trastuzumab, even though the symptoms tended to become better with standard medical care. Patients who received 300 mg/m^2^ or more of doxorubicin or an anthracycline equivalent during therapy or who experienced cardiotoxicity may benefit from echocardiographic follow-up surveillance 1- and 5-years following treatment completion, as recommended by the European Society of Cardiology [84]. Long-term monitoring in elderly and high-risk patients treated with anthracyclines is warranted because of the available evidence showing an increased risk of developing cardiac dysfunction over a 10-year follow-up in patients over 60–65 with colorectal cancer who received adjuvant anthracyclines, even with low-dose anthracyclines [85]. A schematic diagram is shown with the mechanism involved in relation to the cardiotoxicity caused by anthracyclines (Figure 6).

## 5. Management of CRC Anticancer Drug-Related Cardiotoxicity

Many pharmacologic drugs have been demonstrated to have cardioprotective effects in animal studies, but these benefits have not been replicated in human studies of cancer treatment-related cardiotoxicity. Patients receiving anthracyclines or trastuzumab may benefit from cardioprotective medicines such as dexrazoxane, beta-blockers, angiotensin receptor blockers, statins, and aldosterone antagonists [86]. A well-known side effect of chemotherapy drugs is cardiotoxicity. Subclinical injury, overt left ventricular (LV) malfunction, and congestive heart failure (HF) are only a few of the outcomes of cardiotoxicity that can have serious consequences before, during, and after cancer treatment. Primary and secondary prevention are cardio protective measures for high risk patients who have subclinical cardio protective treatments. This review is concerned with the use of cardio protective drugs in conjunction with cancer treatment.

### 5.1. Dexrazoxane

**Figure 7:**
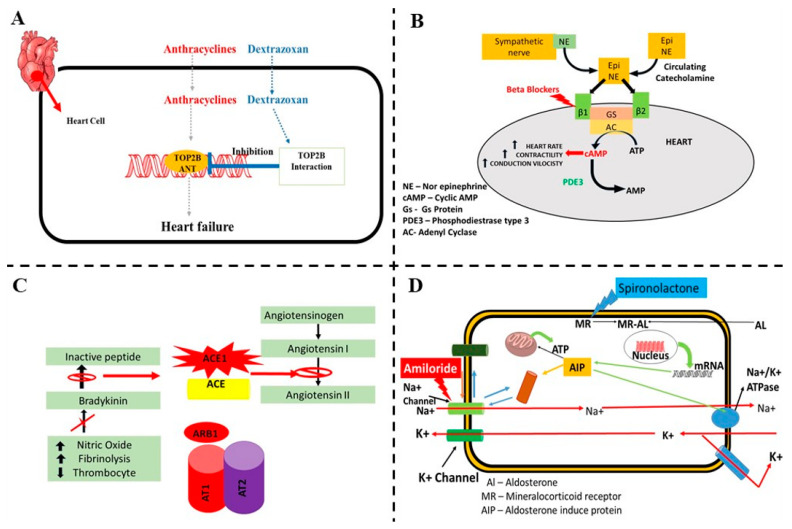
A schematic diagram of the possible mechanisms involved and protection from cardiovascular toxicity associated with colorectal cancer (CRC) drug therapy. (**A**) Anthracycline-induced cardiotoxicity and possible effect of dexrazoxane. (**B**) Beta blockers inhibit the sympathetic activity into cardiac cells and reduce the cardiac toxicity. (**C**) Mechanism involved in relation to the cardiotoxicity caused by anthracyclines and protection by ACE-I and angiotensin receptor blockers. (**D**) Aldosterone antagonist protection in cardiovascular toxicity associated in CRC drug therapy.

Dexrazoxane (DRZ), a bisdioxopiperazine, protects anthracycline- induced heart failure, but its clinical application is constrained by its unclear cardioprotective mechanism, concerns about interfering with anthracycline-induced cancer responses, and worries about long-term safety. The effect of DRZ on the stability of topoisomerases II (TOP2A) and (TOP2B), the DNA damage brought on by the anthracycline, and doxorubicin (DOX) poisoning of these enzymes was studied by Deng et al. (Figure 7A) [87].

DRZ significantly reduces anthracycline-related cardiotoxicity in adults with various solid tumors and children with acute lymphoblastic leukemia and Ewing sarcoma. Numerous studies have demonstrated that the prevalence of HF was reduced among people who used DRZ. Despite these consistent advantages, DRZ has been unable to win over the public. According to ASCO [88], doxorubicin should only be used for cardio protection in patients with metastatic breast cancer who have already received more than 300 mg/m^2^. The iron chelator ethylenediaminetetraacetic acid (EDTA) produces DRZ as a by-product, which can reduce ROS generation. Radical stress, mediated by the superoxide species, is thought to be the primary etiopathogenetic cause of heart damage in the presence of anthracycline. It appears that preventing the formation of anthracycline complexes with metal ions dampens the production of harmful free radicals, hence minimizing cardiac damage, although the precise mechanism by which anthracyclines influence cardiomyocytes is still unknown. Since the 1980s, DRZ has been used in clinical settings as a cardioprotective drug. Concerns about an increased risk of infection, myelosuppression, and secondary malignancies (mostly hematologic), with particular relevance to pediatric use, were raised in a reanalysis of this medication’s safety profile published in 2011 by the European Medicines Agency [89]. Since there was insufficient data showing the drug was safe for use in children and adolescents, it has recommended against their usage.

### 5.2. Beta-Blockers

Beta-blockers inhibit the sympathetic activity into cardiac cells and reduces the cardiac toxicity (Figure 7B). Carvedilol, a nonselective beta-blocker with antioxidant characteristics, is a useful cardioprotective drug to use in conjunction with doxorubicin. It is considered pivotal for the care of HF and LVD patients. The systolic and diastolic functions of the left ventricle (LV) were protected when anthracyclines were used preventatively in a limited group of individuals. Peptide hormone receptor blockers have also been used to modulate RAAS activity. Patients with breast cancer who received locoregional radiation therapy and adjuvant chemotherapy with anthracyclines with or without trastuzumab (n = 120 patients) were enrolled in the PRADA (Prevention of Cardiac Dysfunction during Adjuvant Breast Cancer Therapy) study to prevent LVEF dysfunction [82]. These patients were given a combination of the beta 1 selective adrenergic blocker (B) metoprolol succinate and the angiotensin receptor blocker (ARB) candesartan cilexetil. PRADA was a two-by-two factorial study (beta-blocker vs. ARB or cardioprotective therapy vs. no protective treatment) that used magnetic resonance imaging (MRI) to assess the change in LVEF from the baseline to the end of adjuvant anticancer treatment. Baseline co-morbid diseases or cardiac risk factors were observed in a modest proportion of the research population. Candesartan patients, both those with and without preexisting hypertension, had a 1.8% slower LVEF decline compared to the placebo patients. No significant adjunctive preventive effect of metoprolol on LVEF decrease was seen (*p* = 0.77). There was no proof that the two drugs worked together more effectively [90].

### 5.3. Angiotensin-Converting Enzyme Inhibitors (ACE-I) and Angiotensin Receptor Blockers

In a randomized trial involving 49 patients with a variety of solid tumors, Christian Cadeddu et al. investigated the possible benefit of telmisartan, an angiotensin receptor blocker, in preventing cardiac damage brought on by epirubicin. Twenty-five individuals who started taking telmisartan one week prior to chemotherapy were examined using a tissue Doppler echo technique. The myocardial deformation parameters (peak strain rate) as well as the levels of ROS and interleukin-6 were not statistically different between the patients and the 24 control patients. These findings imply that telmisartan may offer protection against ROS production brought on by epirubicin and may suppress the growth of inflammation, delaying the onset of cardiac damage [91]. Angiotensin-converting enzyme inhibitor (ACE-I) enalapril’s cardioprotective benefits were examined in a randomized, controlled trial involving 473 patients, 53% of whom were being treated with high doses of anthracyclines for breast cancer. A total of 10,014 patients (24%) who displayed an early troponin rise were randomly assigned to either enalapril or no therapy. One month following the end of the chemotherapy, enalapril was started and used for a full year. LVEF remained constant in the enalapril-treated group throughout the observational period [92]. A schematic diagram is shown with the mechanism involved in relation to the cardiotoxicity caused by anthracyclines and the protection by ACE-I and angiotensin receptor blockers (Figure 7C).

### 5.4. Statins

Statins not only reduce LDL cholesterol, but they also have a variety of benefits including anti-oxidative, anti-inflammatory, and others. In an animal model, pretreatment with fluvastatin reduced oxidative stress, increased expression of the antioxidant enzyme mitochondrial superoxide-dismutase-2, and reduced cardiac inflammation caused by anthracycline. Sixty-seven patients with colorectal cancer who were given anthracyclines plus a statin medication were compared to 134 controls in a retrospective case–control study. Statin-treated women had a lower rate of HF at a median follow-up of 2.5 years [93]. A small clinical study found that among the atorvastatin-treated patients, whose LVEF was normal prior to undergoing chemotherapy (which included anthracyclines), the value remained steady after 6 months, but the absolute decline in the controls was 8% [74].

### 5.5. Aldosterone Antagonists

Spironolactone, an aldosterone antagonist, was recently evaluated in a study with 83 breast cancer patients. The patients were randomly randomized to receive either spironolactone or a placebo, both of which were given in conjunction with anthracycline-containing chemotherapy. During at least 24 weeks of treatment including 3 weeks after the conclusion of anthracycline-containing chemotherapy, spironolactone retained diastolic function, lowered the rise in TnI and NT-proBNP, and halted the loss of LVEF [94,95]. A schematic diagram is shown with the mechanism involved in relation to the cardiotoxicity caused by anthracyclines (Figure 7D).

## 6. Conclusions

CRC treatments may have immediate and delayed adverse effects on the heart and blood circulation, exacerbating or masking underlying cardiac conditions. The presence of cardiotoxicity during CRC treatment has the potential to negatively affect primary cancer management by interfering with the timing and optimal doses of cancer therapy intended to cure the disease. Additionally, the creation of a crucial CRC therapy free of malignancy included the idea that cancer patients often have a bad outlook. Involving people at increased risk of a vascular carrying co-existence in clinical trials for agents specifically for acute coronary or arrhythmic events is important for its application in cardiopathy depth analysis. In this review, the different anticancer drugs used in CRC chemotherapy, which induce cardiotoxicity, include trastuzumab, pertuzumab (monoclonal antibodies), lapatinib, sunitinib, pazopanib, sorafenib, imatinib, dasatinib, nilotinib, bosutinib, ponatinib (tyrosine kinase inhibitor), cyclophosphamide, cisplatin, 5-fluorouracil, capecitabine (alkylating agents, fluoropyrimines, fluoropyrimines), vincristine, vinblastine, and vinorelbine (*Vinca* alkaloid) were reviewed based on the available literature (Table 1). Furthermore, the type of cardiotoxicity and the various molecular signaling involved (tyrosine kinase, EGFR, VEGFRs, PDGF, BRAF, mTOR) for promoting the cardiotoxicity in CRC chemotherapy were discussed. 

The field of cardio-oncology research does not benefit from the advancements in medical knowledge and treatments developed for individuals without cancer. Therefore, there is no reason to distinguish between the medical achievements of non-cancer patients and the field of cardio-oncology analysis. Anthracycline-related damage is a primary target of cardiotoxicity drug development, either as preventative measures or for the treatment of individuals who show earlier indicators of internal organ harm that are supported by imaging or biomarkers (e.g., troponin). BCR-ABL1, which belongs to the ErbB family of receptor tyrosine kinases, is used to treat CRC; however, its amplification can lead to a wide range of cellular injury including cardiotoxicity. Myocardial ischemia, smooth muscle hypertrophy, hypertension, and arrhythmias are only some of the cardiac complications that might arise from anticancer drug treatment for CRC treatment. This makes cardiac protection an important aspect during treatment with antineoplastic drugs in CRC therapy. The cytotoxic activities of the molecular signaling inhibitors including anthracycline, tyrosine kinase inhibitor, monoclonal antibodies, etc., are clearly evident. Additionally, these actions may cause cardiotoxicity or cardiovascular disorders. Although the use of cardioprotective measures in the treatment of cardiotoxicity associated with CRC therapy has been suggested, the management of CRC anticancer drug-related cardiotoxicity has to be carefully designed by understanding the molecular pathways involved in various existing cardioprotective drugs like beta blockers, statins, etc. Appropriate methods for managing cardiovascular associated toxicities need to be standardized. In future, molecular signals in triplet therapy-induced cardiotoxicity in individuals with CRC or other cancers might be a serious concern. Therefore, enormous randomized clinical trials need to be performed to understand the CRC associated cardiotoxicity and related multi-signaling pathways to build strong evidence and possible therapies. 

## Figures and Tables

**Table 2:** Cancer therapy-related cardiovascular toxicity definitions.

Symptomatic CTRCD (HF)	Very severe	HF requiring inotropic support, mechanical circulatory support, or consideration of transplantation.
	Severe	HF hospitalization.
	Mild	Mild HF symptoms, no intensification of therapy required.
Asymptomatic CTRCD	Severe	New LVEF reduction to 40%.
	Moderate	New LVEF reduction by ≥10 percentage points to an LVEF of 40–49% OR New LVEF reduction by 10 percentage points to an LVEF of 40–49% AND either new relative decline in GLS by 0.15% from baseline OR new rise in cardiac biomarkers.
	Mild	LVEF ≥50% AND new relative decline in GLS by 0.15% from baseline AND/OR new rise in cardiac biomarkers.

Abbreviations: CTRCD, cancer therapy-related cardiac dysfunction; HF, heart failure; LVEF, Left ventricular ejection fraction; GLS, global longitudinal strain.

## Data Availability

Not applicable.
